# Supplementary material for: Rapid elongation drives the exceptionally fast aggregation of the most common localized human amyloid medin
Source: Commun Chem. 2026 Apr 8;9:194. doi: 10.1038/s42004-026-01950-7 (PMC13237374; doi:10.1038/s42004-026-01950-7)
Supplement: Supplementary file 2 — Supplemental Material [file 42004_2026_1950_MOESM2_ESM.pdf]

SUPPLEMENTARY INFORMATION

Rapid elongation drives the exceptionally fast aggregation of the most common localized human amyloid medin

Vaidehi Roy Chowdhury[1], Robert I. Horne[1], Mariana P. Cali[1], Zenon Toprakcioglu[1], Sara Linse[2] and Michele Vendruscolo[1,*]

[1]Centre for Misfolding Diseases, Yusuf Hamied Department of Chemistry, University of Cambridge, Lensfield Road, Cambridge CB2 1EW, United Kingdom
[2]Biochemistry and Structural Biology, Department of Chemistry, Lund University, SE-221 00 Lund, Sweden

[*]Correspondence to: mv245@cam.ac.uk

Supplementary Notes

Supplementary Note 1.

Kinetic data fitting to protein aggregation models. The integrated rate law equation used for fitting data of an aggregation reaction with an initial monomer mass concentration m_0 and aggregate mass concentration M at any time to a secondary nucleation dominated model is[1]:

$$\frac{M}{M_\infty} = 1 - (1 - \frac{M_0}{M_\infty})e^{-k_\infty t}.(\frac{B_- + C_+e^{\kappa t}}{B_+ + C_+e^{\kappa t}}.\frac{B_+ + C_+}{B_- + C_+})^{\frac{k_\infty}{\kappa \bar{k}_\infty}} \quad (1)$$

where the parameters are defined as

$$\kappa = \sqrt{2m_0k_+m_0^{n_2}k_2} \quad (2)$$

$$\lambda = \sqrt{2k_+k_nm_0^{n_c}} \quad (3)$$

$$C_\pm = \frac{k_+P_0}{\kappa} \pm \frac{k_+M_0}{2m_0k_+} \pm \frac{\lambda^2}{2\kappa^2} \quad (4)$$

$$k_\infty = \sqrt{(2k_+P_0)^2 + \frac{4k_+k_nm_0^{n_c}}{n_c} + \frac{4k_+k_2m_{tot}m_0^{n_2}}{n_2} + \frac{4k_+k_2m_0^{n_2+1}}{n_2+1}} \quad (5)$$

$$\bar{k}_\infty = \sqrt{k_\infty^2 - 2C_+C_-\kappa^2} \quad (6)$$

$$B_\pm = \frac{k_\infty \pm \bar{k}_\infty}{2\kappa} \quad (7)$$

and where

m_0 is the monomer mass concentration at time $t = 0$,

M_0 is the aggregate mass concentration at time $t = 0$ (which is equal to 0 under unseeded conditions),

P_0 is the aggregate number concentration at time $t = 0$ (which is equal to 0 under unseeded conditions),

M_∞ is the mass at infinite time, which is equal to the total protein mass concentration, m_{tot},

k_+ is the elongation rate constant,

k_n is the primary nucleation rate constant,

k_2 is the secondary nucleation rate constant,

k_{off} is the dissociation rate constant,

n_c is the primary nucleation order,
n_2 is the secondary nucleation order.

The off-rate is considered negligible, i.e., $k_{\text{off}} \ll k_+ m_0$.

In the unseeded case, the reaction rate depends only on the combined rate constants $k_+ k_n$ and $k_+ k_2$, not k_+, k_2 and k_n individually. The approximate scaling exponent is:

$$\gamma \approx -\frac{n_2 + 1}{2} \tag{8}$$

The integrated rate law equation used for fitting data of an aggregation reaction to a fragmentation dominated model is[1]:

$$M = M_\infty + Exp\left[-\frac{k_+(4c\kappa\, cosh(\kappa t) + 4P_0\kappa^2\, sinh(\kappa t))}{2\kappa^3}\right]\left((M_0 - M_\infty)e^{\frac{2k_+ c}{\kappa^2}}\right) \tag{9}$$

where the parameters are defined as

$$c = k_n m_0^{n_c} + k_- M_0$$
$$\kappa = \sqrt{2(k_+ m_0 - k_{off})k_-}$$
$$M_\infty = m_{tot} - {k_{off}}/{k_+} \tag{10}$$

and where

k_- is the fragmentation rate constant.

In the unseeded case, for negligible k_{off}, the reaction rate depends only on the combined rate constants $k_+ k_n$ and $k_+ k_-$, not k_+, k_n and k_- individually. The approximate scaling exponent is:

$$\gamma = -\frac{1}{2} \tag{11}$$

Supplementary Note 2.

Comparison of $t_{1/2}$ of aggregation of medin and Aβ42.

$$t_{1/2} \propto m_0$$

Representing $t_{1/2}$ with τ and m_0 with m,

$$\tau = \alpha m^{\gamma} \qquad (\alpha \text{ is a constant})$$

$$\Rightarrow \ln\tau = \ln\alpha + \gamma\ln m$$

(12)

For $\tau_{medin} = \tau_{A\beta_{42}}$,

$$\ln\tau_{medin} = \ln\tau_{A\beta_{42}}$$

$$\Rightarrow \ln\alpha_{medin} + \gamma_{medin}\ln m_{medin} = \ln\alpha_{A\beta_{42}} + \gamma_{A\beta_{42}}\ln m_{A\beta_{42}}$$

(13)

From **Figure 3B**,

$$\ln\alpha_{medin} = -7.4$$

$$\gamma_{medin} = -0.5$$

From **Supplementary Figure 8B**,

$$\ln\alpha_{A\beta_{42}} = -15.9$$

$$\gamma_{A\beta_{42}} = -1.2$$

∴ from (15),

$$\ln m_{A\beta_{42}} = \frac{5\ln m_{medin} - 85}{12}$$

$$\Rightarrow m_{A\beta_{42}} = \frac{(m_{medin})^{5/12}}{1192}$$

(14)

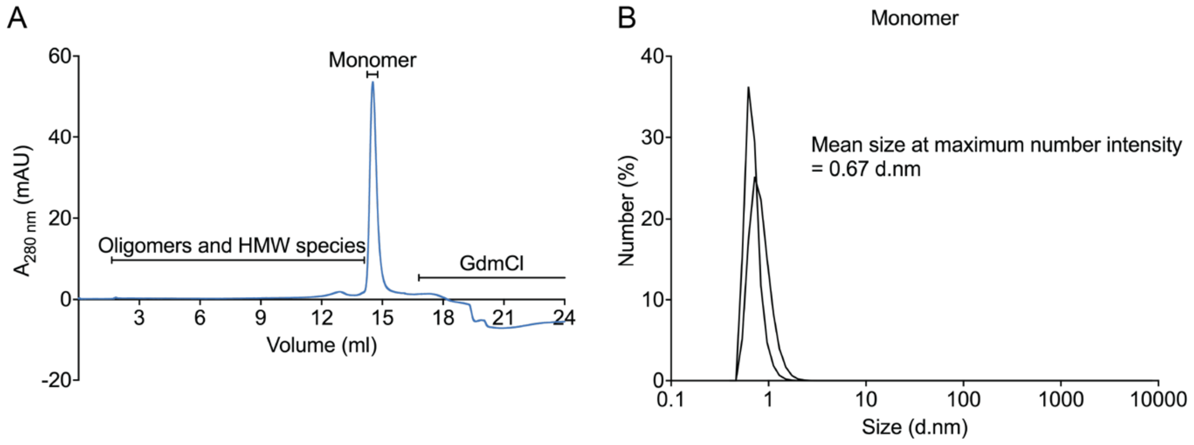

Supplementary Figure 1. Preparation of pure monomer of medin prior to experiments on aggregation kinetics. (A) Medin is denatured in 6 M guanidium chloride (GdmCl) and subjected to size exclusion chromatography. The center of the monomer peak (demarcated by the capped line, 14.25-14.76 mL) is collected in a protein low-binding tube that has been pre-cooled on ice. HMW: high molecular weight. **(B)** Purely monomeric medin diluted to 10 µM in aggregation assay buffer is subjected to DLS. The mean hydrodynamic diameter is found to be 0.67 nm.

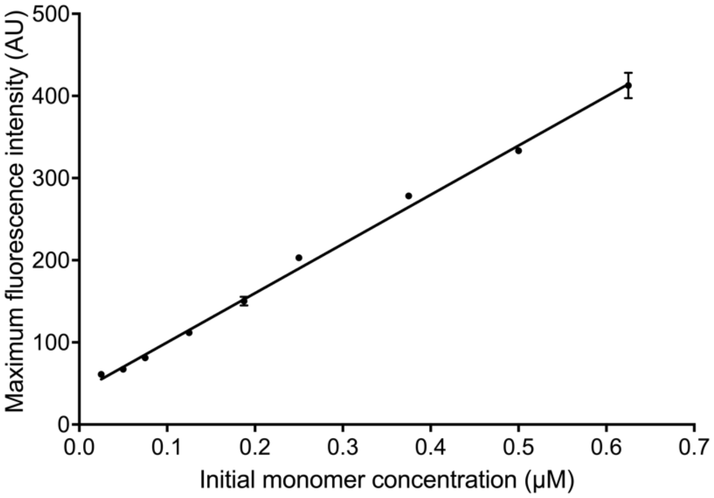

Supplementary Figure 2. ThT fluorescence response against the initial monomer concentration. The maximum fluorescence intensity at the plateau of the sigmoidal curves depends linearly on the initial monomer equivalents required to form fibrils ($R^2 = 0.99$). Data are presented as mean ± SEM.

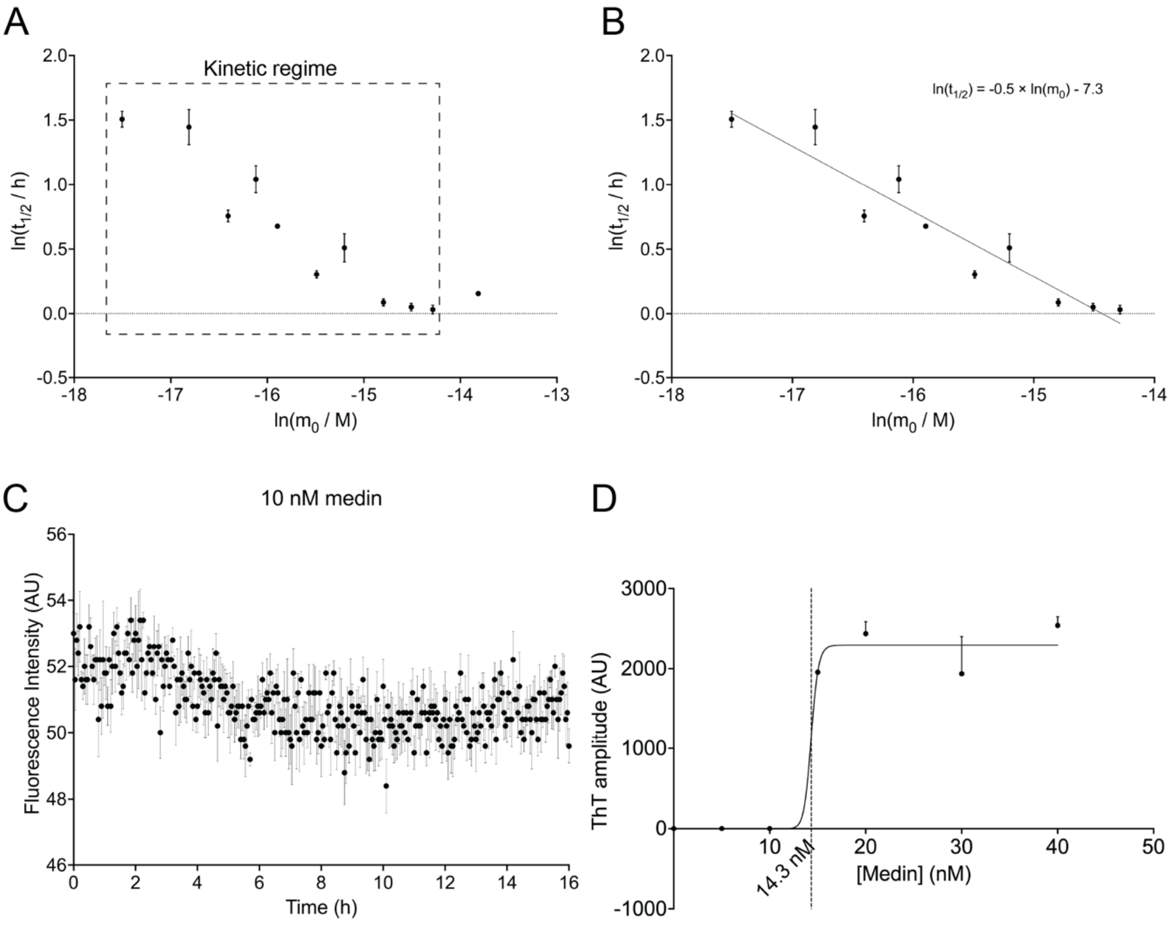

Supplementary Figure 3. Choice of medin concentration range for the determination of the aggregation mechanism. (A) Medin aggregation was monitored at a range of monomer concentrations spanning the physiological range (0-14 μM). The data points shown here correspond to 25-1000 nM. **(B)** A negative scaling of the logarithm of $t_{1/2}$ to the logarithm of m_0 was observed only until 625 nM, which defined the upper boundary of the kinetic regime that we investigated in this work. This range corresponds to the data-points in **(A)** that are enclosed in the dashed-edge box. **(C)** Below 25 nM, e.g. at 10 nM, no aggregation was observed within the reaction timescale. Data are presented as mean ± SEM. **(D)** The critical concentration of medin monomer required to produce a net positive fluorescence emission of ThT at 480 nm above baseline when excited at 440 nm is estimated to be ~14 nM. Data are presented as mean ± SEM.

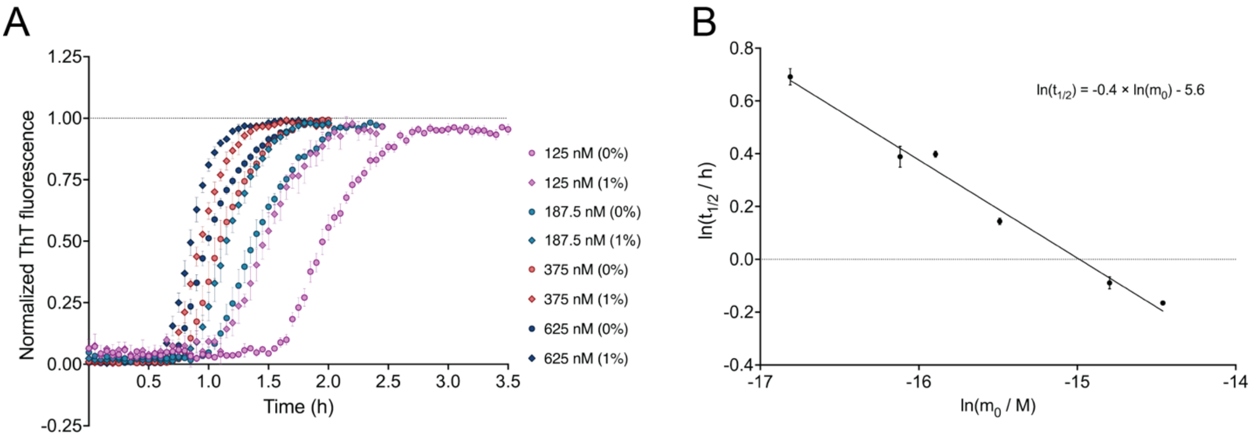

Supplementary Figure 4. Comparison of half-times of unseeded and low-seeded aggregation reactions. (A) Both the t_{lag} and $t_{1/2}$ are reduced when a small quantity of pre-formed fibrils is added as seeds to the aggregation reaction, thus confirming the presence of secondary pathways in medin aggregate nucleation under physiological conditions *in vitro*. Seed:monomer ratios are given in parentheses in the legend. **(B)** Addition of 1% seeds does not significantly alter the mechanism of aggregation nor the weak monomer dependence of nucleation events ($R^2 = 0.96$). Data are presented as mean ± SEM.

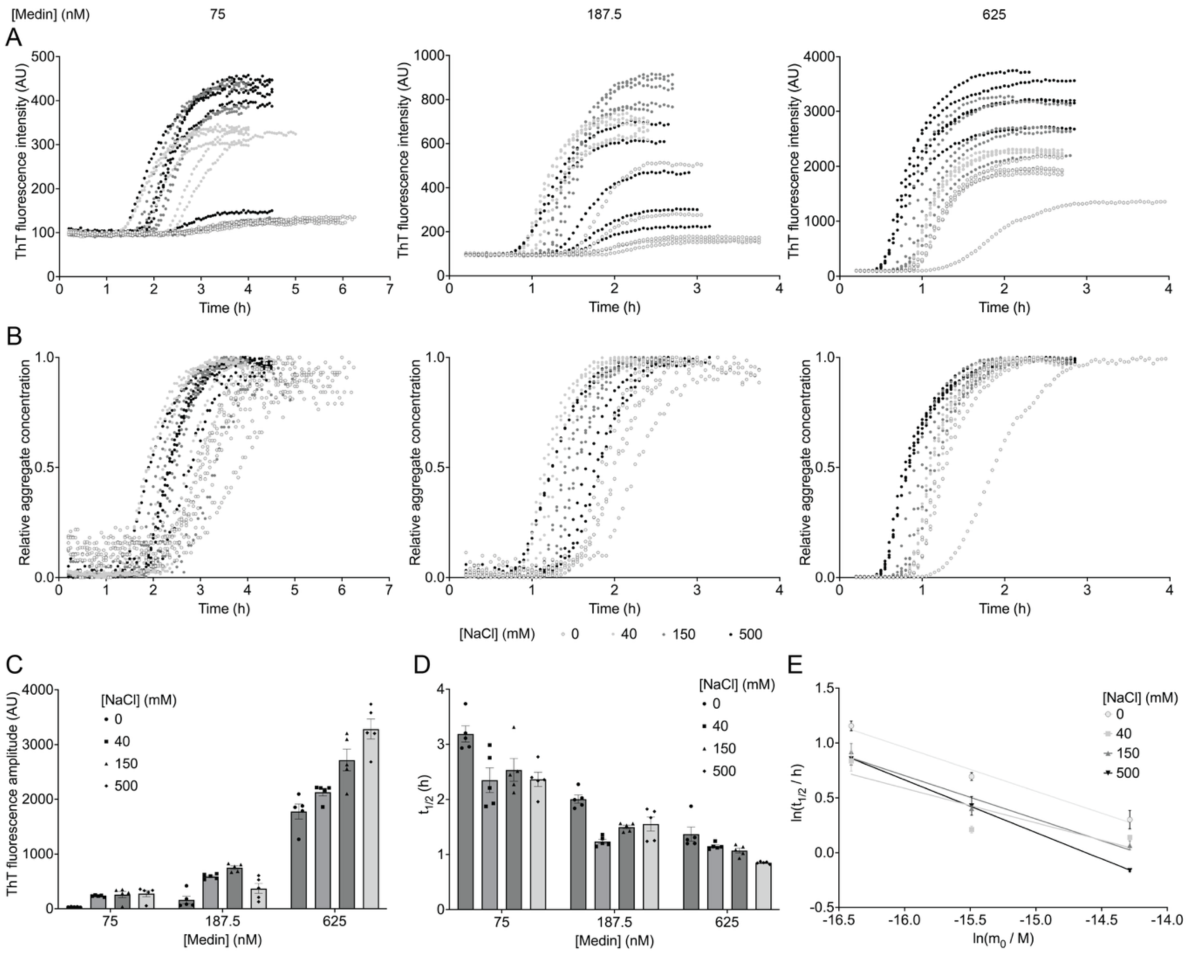

Supplementary Figure 5. Effect of ionic strength on the aggregation kinetics of medin. **(A)** Raw traces and **(B)** normalized profiles of medin aggregation in the presence of increasing concentrations (0, 40, 150 and 500 mM) NaCl monitored using 20 μM ThT are shown. **(C)** The mean amplitude of ThT fluorescence curve is higher in the presence of salt than in the absence thereof. **(D, E)** However, the aggregation half-time does not change appreciably, thereby not affecting the scaling behavior of the $t_{1/2}$ with the m_0. Data are presented as mean ± SEM.

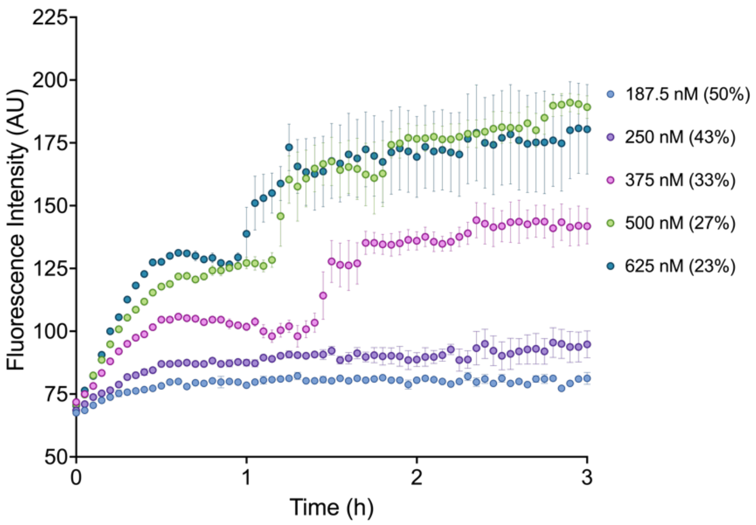

Supplementary Figure 6. Biphasic aggregation profile in high-seeded conditions. Within the initial monomer concentration regime of 375-625 nM in the presence of 187.5 nM seed, there is a typical biphasic aggregation profile, where the reaction stabilizes temporarily before quickly (within 9 min) spiking to a second, higher plateau. Data analysis is done considering the first plateau only. Data are presented as mean ± SEM; AU = arbitrary unit.

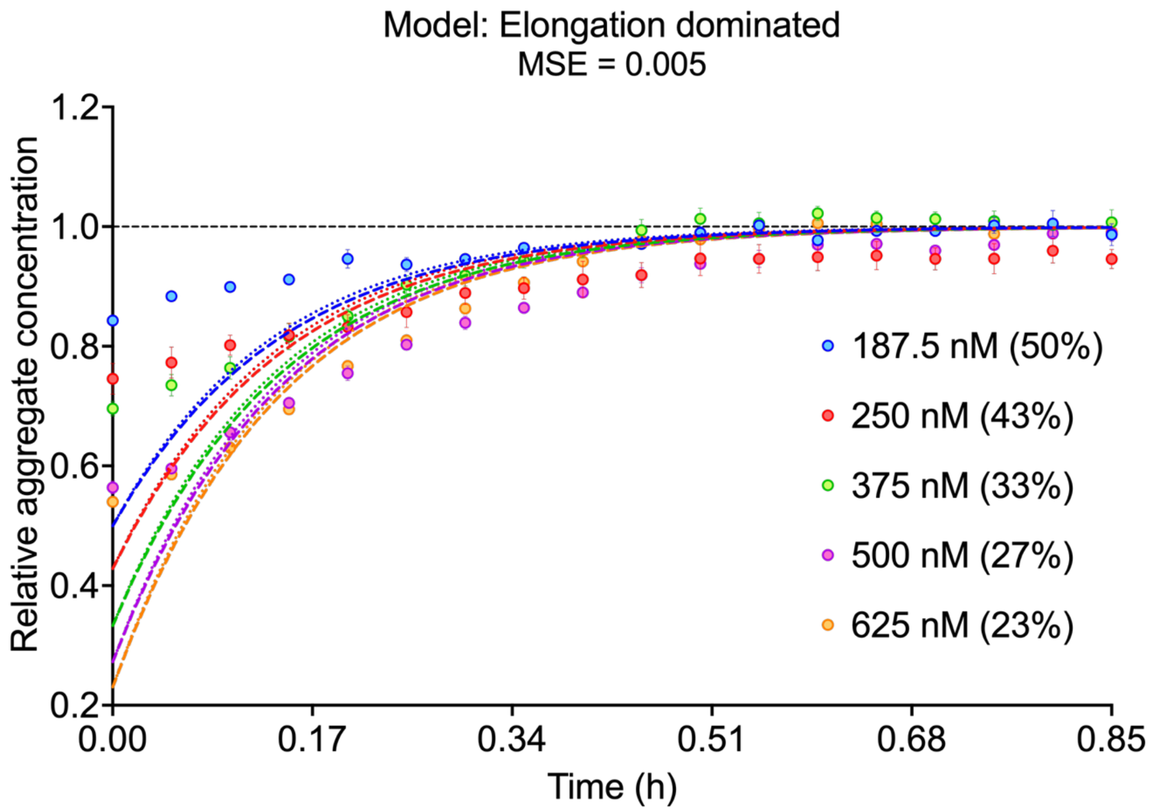

Supplementary Figure 7. High-seeded assays performed with medin monomers (187.5-500 nM) in the presence of high amounts of pre-formed fibrils (187.5 nM; seed:total protein molar ratio mentioned in parentheses) exhibit a characteristic exponential-plateau shape of elongation of existing fibril ends. The normalized data were fitted to negligible rate constants and reaction orders for primary and secondary processes (elongation-dominated model of aggregation) assuming either secondary nucleation (dashed line) or fragmentation (dotted line) dominated nucleation. These assays enabled the determination of the elongation rate constant k_+ to be 5 $\times$ 10^7 M^{-1} s^{-1}.

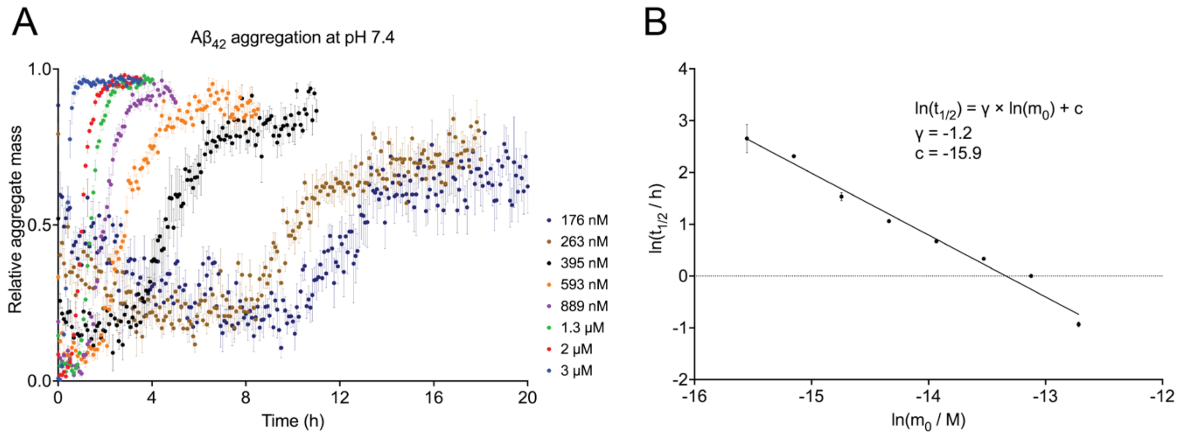

Supplementary Figure 8. Scaling of half-times with initial monomer concentration of Aβ$_{42}$. (A) Recombinant human Aβ$_{42}$ was incubated at 37 °C quiescently in 20 mM NaPi 0.2 mM EDTA pH 7.4 (n = 5). Aggregation was monitored using 20 µM ThT. No aggregation was observed at concentrations below 117 nM within 24 hours. **(B)** The half-time ($t_{1/2}$) of each reaction depends inversely on the initial monomer concentrations (m_0) with a scaling exponent $\gamma = -1.2$, which is characteristic of secondary nucleation ($R^2 = 0.96$). Data are presented as mean ± SEM.

Supplementary Table 1. Sequence analysis of recombinant medin by tryptic digestion liquid chromatography-tandem mass spectrometry (LC-MS/MS). All fragments bigger than 500 Da were detected (unlike previous reports).

Amino acid sequence of tryptic digest product	Mean calculated mass (Da)	Mean observed mass (Da)
QGNFNAWVAGSYGNDQWLQVDLGSSK	2840.312	2840.3181
EVTGIITQGAR	1143.625	1143.6246
NFGSVQFVA	967.9681	967.9684

Supplementary Table 2. Effect of increasing ionic strength on aggregation half-times of medin. ($N = 1$, $n = 5$)

[Medin] (nM)	75		187.5		625	
[NaCl] (mM)	Mean $t_{1/2}$ (h)	SD	Mean $t_{1/2}$ (h)	SD	Mean $t_{1/2}$ (h)	SD
0	3.2	0.3	2.0	0.1	1.4	0.3
40	2.4	0.4	1.2	0.1	1.2	0.0
150	2.5	0.4	1.5	0.1	1.1	0.1
500	2.4	0.3	1.6	0.3	0.8	0.0

Supplementary Table 3. Effect of increasing ionic strength on the scaling exponent (γ) of $\ln t_{1/2}$ to $\ln m_0$.

**[NaCl] (mM)**	**Scaling exponent (γ)**
0	-0.4
40	-0.3
150	-0.4
500	-0.5

Supplementary Table 4. Values of fitted kinetic parameters obtained individually from aggregation assays at high-seeded, low-seeded and unseeded conditions.

Parameter	Symbol	Unit	Model chosen[1]	Mean squared error (MSE)	Fit results
Elongation rate constant	k_+	M^{-1} s^{-1}	Fragmentation dominated	0.00482	5.40E+07
			Secondary nucleation dominated	0.00484	5.34E+07
Secondary pathway rate constant	k_-	s^{-1}	Fragmentation dominated	0.02696	4.26E-08
	k_2	s^{-1}	Secondary nucleation dominated	0.02688	4.36E-08
Secondary nucleation Order	n_2	Unitless	Secondary nucleation dominated	0.02688	~0
Combined rate constant	k_+k_n	$M^{-1}s^{-2}$	Fragmentation dominated, no seed	0.031	3.30E-01
			Secondary nucleation dominated, no seed	0.039	2.31E-02
Primary nucleation order	n_c	Unitless	Fragmentation dominated, no seed	0.031	~1
			Secondary nucleation dominated, no seed	0.039	~1

Supplementary Table 5. Values of fitted kinetic parameters from global data analysis. The MSE for both the fragmentation- and secondary nucleation-dominated models is 0.02.

**Parameter**	**Symbol**	**Unit**	**Model chosen**[1]	**Fit results**
Elongation rate constant	k_+	M^{-1} s^{-1}	Fragmentation dominated	9.53E+06
			Secondary nucleation dominated	9.46E+06
Secondary pathway rate constant	k_-	s^{-1}	Fragmentation dominated	3.14E-07
	k_2	s^{-1}	Secondary nucleation dominated	3.23E-07
Secondary nucleation order	n_2	Unitless	Secondary nucleation dominated	~0
Primary nucleation rate constant	k_n	s^{-1}	Fragmentation dominated	1.15E-08
		s^{-1}	Secondary nucleation dominated	1.13E-08
Primary nucleation order	n_c	Unitless	Fragmentation dominated	~1
			Secondary nucleation dominated	~1

Supplementary Table 6. Model verification over biological replicates in unseeded assays ($N = 3$).

**Model chosen**[1]	**Parameter**	**Symbol**	**Unit**	**Average value**	**Standard deviation**
Secondary nucleation dominated, no seed	Combined rate constants	k_+k_n	$M^{-1}s^{-2}$	0.1	0.1
		k_+k_2	$M^{-1}s^{-2}$	22.3	20.1
	Primary nucleation reaction order	n_c	Unitless	~1	0.1
	Secondary nucleation reaction order	n_2	Unitless	~0	0.1
Fragmentation dominated, no seed	Combined rate constants	k_+k_n	$M^{-1}s^{-2}$	7.3	6.6
		k_+k_-	$M^{-1}s^{-2}$	1.7	0.5
	Primary nucleation reaction order	n_c	Unitless	1.4	0.1

Supplementary References

1. Meisl G, Kirkegaard JB, Arosio P, Michaels TC, Vendruscolo M, Dobson CM, Linse S, Knowles TP: **Molecular mechanisms of protein aggregation from global fitting of kinetic models.** *Nat Protoc* 2016, **11:**252-272.
