# Supplementary material for: Rapid elongation drives the exceptionally fast aggregation of the most common localized human amyloid medin
Source: Commun Chem. 2026 Apr 8;9:194. doi: 10.1038/s42004-026-01950-7 (PMC13237374; doi:10.1038/s42004-026-01950-7)
Supplement: Supplementary file 3 — Description of Additional Supplementary Files [file 42004_2026_1950_MOESM3_ESM.pdf]

Description of Additional Supplementary Files

File name- Supplemental Data

File description – All data pertaining to the manuscript (Main and Supplementary Information) have been provided in the Supplementary Data file.
